# Arbuscular and Ectomycorrhizal Fungi Associated with the Invasive Brazilian Pepper Tree (*Schinus terebinthifolius*) and Two Native Plants in South Florida

**DOI:** 10.3389/fmicb.2017.00665

**Published:** 2017-04-20

**Authors:** Karim Dawkins, Nwadiuto Esiobu

**Affiliations:** Microbial Biotech Lab, Biological Sciences Department, Florida Atlantic UniversityBoca Raton, FL, USA

**Keywords:** arbuscular mycorrhiza, ectomycorrhiza, plant invasion, rhizobiome, biotic resistance, Brazilian pepper tree

## Abstract

The potential role of soil fungi in the invasion of the Brazilian pepper tree (*Schinus terebinthifolius*—BP) in Florida is not known; although the low biotic resistance of Florida soils is often invoked to explain the prevalence of many invasive species. To gain an initial insight into BP's mycorrhizal associations, this study examined the rhizobiome of BP and two native plants (*Hamelia patens* and *Bidens alba*) across six locations. Arbuscular mycorrhizal fungi (AMF) associated with the roots of the target plants and bulk soil was characterized by spore morphotyping. Sequence analysis of metagenomic DNA from lateral roots/rhizosphere of BP (*n* = 52) and a native shrub *H. patens* (*n* = 37) on the same parcel yielded other fungal associates. Overall, the total population of AMF associated with BP was about two folds greater than that of the two native plants (*p* = 0.0001) growing on the same site. The dominant AMF under *Schinus* were members of the common *Glomus* and *Rhizophagus* spp. By contrast, the most prevalent AMF in the bulk soil and rhizosphere of the two Florida native plants, *Acaulospora* spp (29%) was sharply diminished (9%) under BP rhizosphere. Analysis of the ITS2 sequences also showed that *Schinus* rhizosphere had a high relative abundance of ectomycorrhizal fungi (76.5%) compared to the native *H. patens* (2.6%), with the species *Lactifluus hygrophoroides* (Basidiomycota) being the most prevalent at 61.5% (*p* < 0.05). Unlike the native plants where pathogenic fungi like *Phyllosticta* sp., *Phoma* sp., and *Neofusicoccum andium* were present (8.1% for *H. patens*), only one potentially pathogenic fungal taxon was detected (3.9%) under BP. The striking disparity in the relative abundance of AMF and other fungal types between BP and the native species is quite significant. Fungal symbionts could aide plant invasion via resource-use efficiency and other poorly defined mechanisms of protection from pathogens in their invaded range. This report exposes a potentially significant but previously unappreciated fungal dimension of a complex invasion system and underscores the need to characterize these fungal symbionts, their role and mode of action during invasion; with the goal of devising measures for invasion control and ecological restoration.

## Introduction

The intricately complex mechanisms and processes underlying plant invasion in non-native regions include enemy release, resource use efficiency and the novel weapons hypotheses among others (Callaway et al., 2004, 2008; Rai, 2013; Dawkins and Esiobu, 2016). Several studies have addressed the effects of climate change, enemy release, and many other hypotheses on plant invasion, but very little is known about the concept of biotic resistance and how it contributes to making one soil more susceptible to plant invasion than others (Levine et al., 2004). Biotic resistance refers to the ability of native species to reduce the success of exotic invasive plants through predation, herbivory, and soil microbial antagonism (Levine et al., 2004). It has been shown for many other invasive species that the release from pathogens in their native habitat coupled with improved resource use efficiency can enhance their establishment and spread (Blumenthal et al., 2009). The native biotic and abiotic environment play important roles in plant invasion but of equal significance is the propensity of the invasive plant to change the direction of soil feedback from negative to positive (Klironomos, 2002). Native plants are said to accumulate a more negative soil feedback due to accumulation of more root pathogens and parasites. Positive soil feedback exhibited by invasive plants in non-native regions occurs by the accumulation of mutualistic symbionts and enemy release (Reinhart and Callaway, 2006). It may then be inferred that areas with low biotic resistance may have a neutral to weakly negative soil feedback which would allow many invasive species to establish and spread (Maron and Vila, 2001). The Brazilian pepper tree (*Schinus terebinthifolius*) is a dominant invasive plant in Florida and Hawaii (sub-tropical regions) but has not been as aggressive and widespread in Texas and California (Hight et al., 2003). Many other studies have elucidated some invasive mechanisms of plants, including physical/evolutionary environment adaptation (Spector and Putz, 2006; Geiger et al., 2011), resource use efficiency (Carneiro et al., 1996; Ewe and Sternberg, 2002) and enemy release mechanisms (Rai, 2013). Other emerging strategies for invasion include the disruption of soil microbial communities by invasive plants which allow their progression while negatively impacting native plants. These emerging mechanisms need to be studied for the Brazilian pepper tree to provide insight which could improve control measures and help boost ecological restoration efforts across Florida and other affected states. The soil fungal community serves many beneficial roles in the rhizosphere of plants, forming a unique symbiosis (Aziz et al., 1995). Arbuscular mycorrhizal fungi (AMF) from the Glomeromycota and ectomycorrhizae are two common or widely known plant-microbe symbionts in soil (Hibbert et al., 2000; Redecker and Raab, 2006; Smith and Read, 2008). Invasive plants such as *Pinus* spp. and *Centaurea maculosa* have been shown to take advantage of these usually widespread beneficial fungi (Richardson et al., 1994; Lekberg et al., 2013) during invasion. Another key element possibly involved in plant invasion, is the association of plants with common mycorrhizal networks (Smith and Read, 2008; Barto et al., 2012). It has been reported that certain plants associate with some non-host specific mycorrhiza, employing the fungal hypha to transport allelochemicals to nearby co-occurring plants inhibiting their growth (Barto et al., 2011). Interestingly, it is not uncommon to find monocultures of *Schinus* trees displace previously diverse flora in Florida by mechanisms not well understood. Although no allelochemicals have yet been implicated in *Schinus* invasion; it has been shown to make a range of antimicrobials and seed germination inhibitors (Morgan and Overholt, 2005; Alves et al., 2013).

In an unrelated study with the Brazilian pepper tree seeds, the authors found consistent fungal “contaminants” on the seeds even after routine surface sterilization (Shetty et al., 2011). And since there is scarcely any published literature on fungal symbionts or associates of this aggressive invasive plant in Florida, we designed this brief study to characterize its rhizosphere fungal community structure and compare same with two Florida natives found in the vicinity. The goal was to examine the extent to which the introduced plant species is associated with (a) ectomycorrhiza, (b) potentially useful arbuscular mycorrhiza, and (c) known fungal pathogens. We hypothesized that the fungal community structure of Brazilian pepper tree rhizosphere will contain a high proportion of potentially beneficial fungi and fungal network; and less pathogenic ones compared to native plants. This analysis will hopefully provide clues into the abundance of fungal groups occurring under the Brazilian pepper tree rhizobiome that could directly or indirectly enhance invasion of its non-native range.

## Materials and methods

### Rhizosphere and soil sampling

Six sampling sites (Table 1) with at least 1 year history of Brazilian pepper (*Schinus terebinthifolius*) invasion, presence of dominant or monoculture stands of Brazilian pepper trees; and the co-existence of two Florida native plants in adjacent (~600 m) parcels of land were chosen across Southeastern Florida; including Palm Beach, Broward and Miami-Dade counties. The two Florida native species, Shepherd's needle (*Bidens alba*) and Firebush (*Hamelia patens*) were chosen as the non-invasive plant species whose rhizosphere fungal communities were contrasted with that of the Brazilian pepper tree. Shepherd's needle from the family Asteraceae is an annual or perennial short lived herbaceous weed which in a previous study (Morgan and Overholt, 2005) was shown to be inhibited by Brazilian pepper tree leaf extracts. Firebush is a fast growing perennial shrub plant from the family Rubiaceae which is found extensively throughout Southeastern Florida. Although these plants are phylogenetically unrelated to the invasive species from the family Anacardiaceae, they were the ones found consistently on the same parcel of land (sampling site) as the Brazilian pepper tree. Their individual rhizosphere community structure compared to that of the bulk soil should illustrate their relative response to and interaction with the fungi in that particular site. At each sampling site, 3 dominant stands of Brazilian pepper tree (>1 year old) were identified and sampled at a depth of 15–20 cm where roots and adjacent soil were removed. For each Brazilian pepper stand, three rhizosphere samples were collected by digging up roots with surface sterilized shovels; and then vigorously shaking off of the soil (approximately 100 g) into a sampling bag; to make one composite sample (Rep 1) using a similar protocol (Inceoglu et al., 2010). The process was repeated twice to produce three *S. terebinthifolius* replicates for each site, yielding a total of 18 composite rhizosphere samples. The replicates were sampled at least 10 m apart from each other. The native plants were sampled within a ~600 m radius using the same protocols. As a control, bulk soil without plant cover was sampled at the same depth using an improvised sterile soil borer of 15 cm in length by a 3 cm diameter. Three to five centimeters of top soil was first removed to eliminate litter and debris before samples were collected. The total number of samples collected was seventy-two (72) [(3 Reps × 3 Plants × 6 sites) + (3 Bulk × 6 Reps)].

**Table 1:** **Sample metadata collected from the six sampling sites throughout South-Eastern Florida**.

Sample sites^*^	**County**	**BP invasion history**	**Soil type**	**Key features**
Site 1				
Tree Tops Park	Broward	>15 years	Sandy/loamy	Multiple dominant stands
26° 4′ 18″N, 80° 16′ 35”W				
Site 2				
W Atlantic Ave/Lyons Rd.	Broward	Unknown	Sandy/loamy	Multiple dominant stands
26° 14′ 5.6″N, 80° 11′ 16″W				
Site 3				
West Delray Regional Park	Palm Beach	>15 years	Sandy/loamy	Few dominant stands adjacent to a pond
26° 27′ 41″N, 80° 13′ 10.8″W				
Site 4				
Dyer Park	Palm Beach	>15 years	Sandy/loamy	Few dominant stands adjacent to a pond
26° 47′ 19″N, 80° 7′ 22″W				
Site 5				
R Hardy Matheson Preserve	Miami-Dade	>15 years	Sandy/loamy	Few dominant stands
25° 39′ 24″N, 80° 16′ 48″W				
Site 6 Φ				
Oleta River State Park	Miami-Dade	>15 years	Coarse sandy	Very few dominant stands (withering - BP)
25° 55′ 0.3″N, 80° 8′ 19″W				

* *Samples were collected between March and May 2015*.

^*^Rainfall and temperature data were consistent throughout the different sampling periods.

Φ *On-going physical and chemical control measures to eradicate BP was observed*.

### Enumeration and identification of arbuscular mycorrhizal spores

Arbuscular mycorrhizal fungal (AMF) spores from each sample (*n* = 72), was enumerated by a modified method (Gerdemann and Trappe, 1974; Pacioni, 1992). Ten grams (10 g) of the soil and 2 g of root crushed by mortar and pestle were suspended in 100 mL of sterile distilled/deionized water followed be a sucrose gradient extraction. The supernatant was then decanted into two 3″ USA standard sieves of decreasing pore size 125 and 32 μm mesh (W. S. Tyler Industrial Group, OH, USA). The filtered content on both sieves was then rinsed with sterile water, dried and viewed using the Leica Zoom 2000 stereomicroscope (Leica Microsystems Inc., IL, USA) at 675X total magnification. The total # of fields of view was calculated (208) and multiplied by the average number of spores seen in at least 30 fields of view to enumerate the number of spores per gram (Bever et al., 1996). Data from the International Culture Collection of Vesicular Arbuscular Mycorrhizal fungi (INVAM) (Perez and Schenck, 1989) which consist of numerous classified AMF spore information was used to determine the presumptive Genus of a subsample of AMF extracted from *S. terebinthifolius*, the Florida native plants and bulk soil based on their size, pigmentation and morphology. After the differential counts were obtained, relative abundance (%) for each AMF type was computed based on the total numbers for the same sample.

### Determination of soil pH

A subsample of soil from the original sample bag was removed to produce a 1:1 suspension of soil and distilled water (10 g soil: 10 ml water) mixed every 10 min over a 30 min period. A calibrated Sartorius PB-11 pH meter (Sartorius Corp., NY, USA) was used to measure the pH of each soil sample after 1 min (Soil Survey Staff, 2014).

### Metagenomic DNA extraction

Only rhizosphere samples from the invasive shrub—*S. terebinthifolius* and the native shrub *H. patens* were employed in this section. Using a modified protocol from the MoBio powersoil DNA kit (Mo Bio Laboratories Inc., CA, USA), 2 g of rhizosphere soil samples with lateral roots from *S. terebinthifolius* and *H. patens* were first gently ground using a mortar and pestle to help soften the rigid chitin rich fungal cell walls and immersed in 8 ml of extraction buffer. After high speed centrifugation, the cell pellets were then subjected to the MoBio powersoil kit and protocol according to manufacturer instructions. Extracted DNA was quantified using the Nanodrop 2000c spectrophotometer (Thermo Fisher Scientific, MA, USA) and run on a 1% agarose gel (1X TAE) to visually verify the integrity of the DNA samples.

### Amplification of the fungal internal transcribed spacer (ITS2)

The ITS2 region was amplified using the ITS86F (5′-GTGAATCATCGAATCTTTGAA-3′) and ITS4R (5′-TCCTCCGCTTATTGATATGC-3′) universal primers (Vancov and Keen, 2009). The 2X Promega PCR master mix (Promega Corp., WI, USA) was diluted two-fold along with 0.4 μM of each primer, 10 μg bovine serum albumin (Promega Corp., WI, USA) and 2 mM MgCl_2_ and ~20 ng of DNA template for each sample in a final 25 μl reaction mix. PCR amplification was carried out using an Eppendorf gradient thermocycler (Eppendorf, Hamburg, Germany) with an initial denaturation of 94°C for 3 min, followed by 34 cycles of 94°C for 45 s, 55°C for 45 s and 72°C for 2 min. For samples that did not amplify at this annealing temperature a gradient PCR was used between 54.2 and 52.1°C annealing temperatures to lower the stringency of the primer-template binding. A final extension step was carried out at 72°C for 7 min. The PCR reactions were done in triplicate for each sample and then pooled to obtain a more representative portion of the soil fungal community.

### Transformation and cloning of ITS-PCR products

The TOPO® TA Cloning kit for sequencing (Thermo Fisher Scientific Inc., MA, USA) was used to splice in the Taq polymerase amplified ITS-PCR products into the pCR™ 4-TOPO vector. Recombinant plasmids were transformed into competent DH5-α *E. coli* cells following manufacturer's instructions. True clones (white colonies) bearing individual ITS2 amplicons were then picked, sub-cultured on fresh LB with 100 μg/ml ampicillin and incubated for 24 h at 37°C before plasmid extraction using the Zippy plasmid mini-prep kit (Zymo Research Corp., CA, USA).

### Sequence analysis

Out of 162 clones submitted for sequencing, eighty nine (89) recombinant plasmid extracts (*n* = 52 for *S. terebinthifolius, n* = 37 for *H. patens*) were successfully sequenced and analyzed. (Table 2) by Eurofins MWG Operon using the T3 forward primer (5′- GCAATTAACCCTCACTAAAGG-3′) recommended by the pCR4 TOPO kit manufacturer (Thermo Fisher Scientific Inc., MA, USA). Vector contamination was first removed from the FASTA sequence files using the Lucy vector trimming tool. The RDP Naïve Bayesian rRNA classifier version 2.10 (Wang et al., 2007) was used to classify sequences using the “Warcup” training set of Internal Transcribed Spacer sequences database (Deshpande et al., 2015) for fungal ITS sequences and NCBI BLAST using the nucleotide collection database. Sequences were aligned using the Clustal Omega multiple sequence alignment tool. Representative ITS2 sequences from *S. terebinthifolius* and *H. patens* rhizosphere were deposited in Genbank with accession numbers KX816625-KX816643 and KY228415.

**Table 2:** **Distribution of sequenced clones across the six sample sites**.

**Sample site/description**	***S. terebinthifolius* Total # of plasmid clones (#sequenced)**	***H. patens* Total # of plasmid clones (#sequenced)**
Site 1	20 (11)	13 (8)
Tree Tops Park		
Site 2	9 (2)	11 (6)
W Atlantic Ave/Lyons Rd.		
Site 3	11 (4)	9 (3)
West Delray Regional Park		
Site 4	19 (10)	5 (1)
Dyer Park		
Site 5	28 (20)	8 (5)
R Hardy Matheson Preserve		
Site 6	8 (5)	21 (14)
Oleta River State Park		
Total	95 (52)	67 (37)

### Statistical analysis

To test the statistical significance of the observed differences in AMF spore counts and the different morphological types of AMF, a two way ANOVA was done. The site and plant effects, along with interactions in bulk soils, BP and the native plants were tested at the 95% confidence limit followed by a Tukey HSD *post-hoc* test. Molecular data was subjected to an unpaired *T*-test to assess the potential effect of observed trends of ectomycorrhizal prevalence under BP, and the Florida native shrub (*H. patens*) by grouping the six sites into three counties (soil types—Table 1).

## Results

Unlike the bulk soil and the Florida native plants (*Hamelia patens* and *Bidens alba*) whose rhizosphere soil pH ranged from 6.57 to 7.35; the average pH of the Brazilian pepper tree rhizosphere was slightly acidic (6.36). The pH at site #6 was the highest (7.8) and was significantly (*p* < 0.05) higher than other sites for *S. terebinthifolius* and the two native plants.

**Figure 1:**
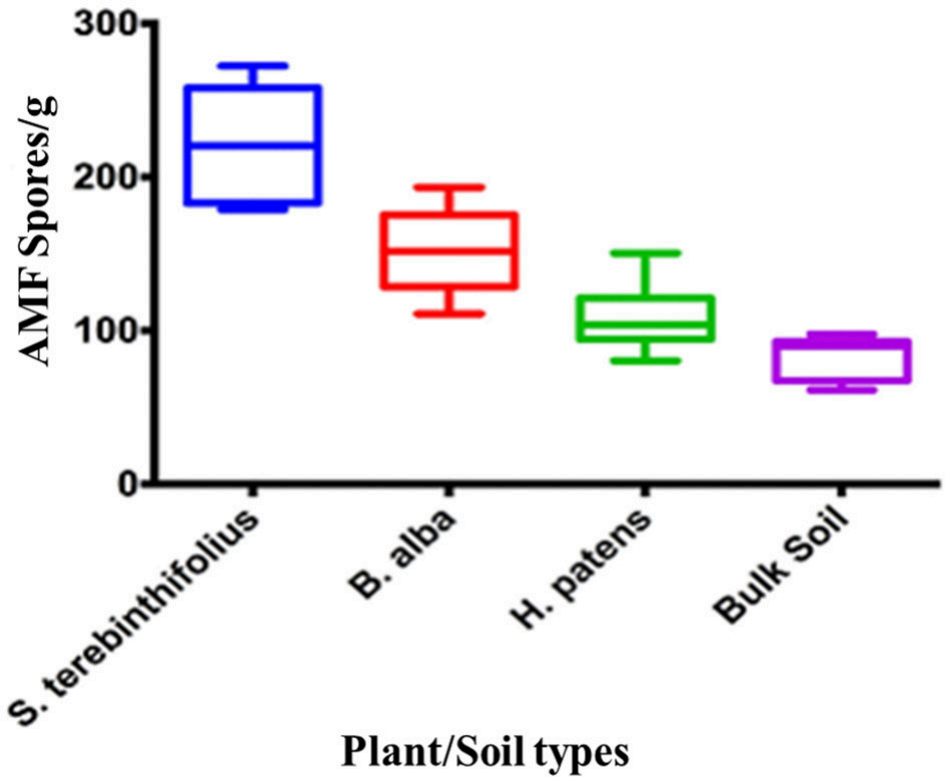
**Numerical abundance of AMF spores extracted from ***S. terebinthifolius***, ***H. patens***, and ***B. alba*** rhizospheres and bulk soil in six south Florida locations**.

Arbuscular mycorrhizal fungal spores were almost two times more abundant in the *S. terebinthifolius* rhizosphere than in those of the Florida native plant (Figure 1, Table S1). Prevalence ranged from 65 spores/g (bulk soil) to 275 spores/g for *S. terebinthifolius*.

Table 3A displays the average AMF population associated with *S. terebinthifolius* rhizosphere, *H. patens* and *B. alba*. The abundance of these beneficial fungi under the BP were significantly higher than the bulk soil and the native plants using a two way ANOVA and confirmed with the *post-hoc* Tukey HSD test (Tables 3A,B). There was also an observed site effect found with for sites 3, 5, and 6 (*p* = 0.00149) along with many site:plant interactions (*p* = 0.01318). Soil attributes, including vegetation type, pH and other resident organisms could have affected the spore counts in the rhizosphere samples.

**Table 3A:** **Average Counts of arbuscular mycorrhizal fungal (AMF) spores extracted from bulk soil and the rhizosphere of ***S. terebinthifolius*** and two Florida native plants (***H. patens*** and ***B. alba)*** from six sampling locations**.

**Sample sites/county**	**Bulk soil (mean ± SE)**	S. terebinthifolius^*^ (mean ± SE)	***H. patens* (mean ± SE)**	***B. alba* (mean ± SE)**	Site average^**^ (mean ± SE)
Site 1/Broward	69.3 ± 20	237.8 ± 79	111.3 ± 39	134.5 ± 25	138.2 ± 70.2^b^
Site 2/Broward	90.1 ± 13	253 ± 47	101.3 ± 5.9	169.1 ± 53	153.4 ± 74^b^
Site 3/Palm Beach	91.5 ± 18	272.3 ± 53	106.1 ± 14	193.2 ± 6.8	165.8 ± 82^ab^
Site 4/Palm Beach	97.7 ± 24	178.8 ± 15	150.4 ± 38	156 ± 36	145.7 ± 34.3^b^
Site 5/Miami-Dade	61.4 ± 12	184.4 ± 22	99.1 ± 19	147.5 ± 9.2	123.1 ± 53^bc^
Site 6/Miami-Dade	90.1 ± 19	203.1 ± 28	80.4 ± 11	110.9 ± 18	121.1 ± 55^bcd^
Averages counts	83.3 ± 12	221.6 ± 31	108.1 ± 19	151.9 ± 23	

* *Significant plant effect (p = 2e-16) was observed for S. terebinthifolius compared to the natives*.

** *Significant site effect (p = 0.00149) was observed for Sites 3 and 5 and Sites 3 and 6*.

**Column means followed by the same letter are not significantly different by the Tukey HSD test (p < 0.05).

**Table 3B:** **Results of two way ANOVA and Tukey HSD ***post-hoc*** statistical analysis of the effects and interactions of sample site and plant type on the prevalence of AM spores**.

	**Df**	**Sum sq**	**Mean sq**	***F*-value**	**Pr (>F)**
Site	5	18,130	3,626	4.668	0.00149
Plant type	3	198,239	66,080	85.070	2e-16
Site: PlantType	15	27,276	1,818	2.341	0.01318
Residuals	48	37,285	777		
**Significant site effect**	**Adjusted** ***p*****-value**	**Significant plant effect**		**Adjusted** ***p*****-value**	
Site 3 and 5	0.0060277	*Schinus*:*Bidens*		0.0000001	
Site 3 and 6	0.0035800	*Schinus*:*Hamelia*		0.0000001	

**Figure 2:**
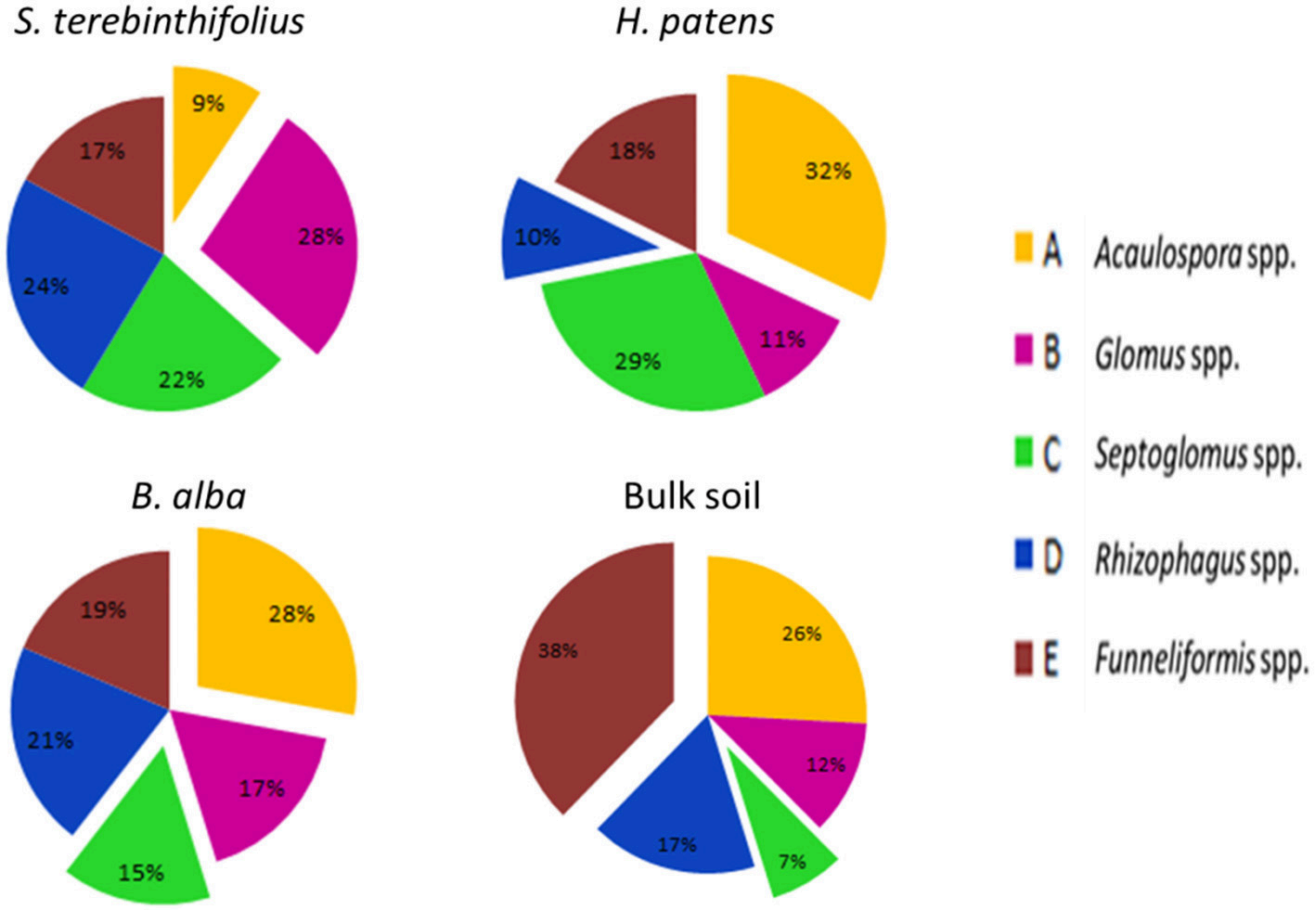
**Relative abundance of various types of morphologically distinct arbuscular mycorrhizal spores recovered from the rhizosphere of the invasive plant ***S. terebinthifolius***, natives (***H. patens*** and ***B. alba***) and bulk soil in Broward county**. The relative abundance of *Glomus* spp and *Rhizophagus* spp. under *S. terebinthifolius* found to be significantly different than other plant species and bulk soil (*p* < 0.05).

In Table 4A, the average counts of the different types of AMF extracted from the rhizosphere of the native plants, the invasive species and the bulk soil are presented. With the exception of the *Acaulospora*, spp and *Funneliformis* spp; the counts of all other AMF were higher in the BP rhizosphere than that of the native plants and the bulk soil. Notably, the prevalence of *Glomus* spp. and *Rhizophagus* spp. was significantly higher under BP rhizosphere, confirmed by the Tukey HSD test (Tables 4A–C). Figure 2 illustrates the sharp contrast in the relative abundance and community structure of AMF between the *S. terebinthifolius* on one hand, and the two native plants and bulk soil on the other hand. Arbuscular mycorrhiza of the A -*Acaulospora* spp (Light brown, clear—translucent globose 50–100 μm diameter) constituted 28–32% and only 9% of AMF for native plants and *S. terebinthifolius* respectively. The genus—*Glomus* spp. was morphotyped as (B—Brown, ellipsoid and translucent >125 μm spores). The C and D spore types were differentiated as *Septoglomus* spp. (brown/black, globuse, 50–100 μm) and *Rhizophagus* spp. (clear/light brown, subglobuse, 50–100 μm) respectively. Interestingly, *Funneliformis* spp., spore type E (brown, opaque, globose >125 μm), was uniformly present in all the plant types—natives and invasive at ~17.5% compared to 38% in bulk soil (Figure 2, Table S2).

**Table 4A:** **Morphotypes of AMF spores (average) extracted from bulk soil, the rhizosphere of invasive ***S. terebinthifolius*** and Florida natives (***H. patens*** and ***B. alba***) from two sample sites (Broward County) using the INVAM classification**.

**AMF genus**	**Spore description**	**Designated codes**	***S. terebinthifolius* (Mean ± SE)**	***B. alba* (Mean ± SE)**	***H. patens* (Mean ± SE)**	**Bulk soil (Mean ± SE)**
*Acaulospora* spp.	Light brown, globose, clear—translucent, 50–100 μm	A	4 ± 1	6 ± 3	5 ± 0	3 ± 3
Glomus ^*^spp.	Brown, ellipsoid, translucent, >125 μm	B	9 ± 2^ab^	3 ± 0^b^	2 ± 1^b^	1 ± 0^b^
Septoglomus spp.	Brown/black, globose/ subglobose, 50–100 μm	C	2 ± 1	1 ± 2	1 ± 1	0
Rhizophagus ^**^spp.	Clear/light brown, subglobose, 50–100 μm	D	6 ± 1^ab^	2 ± 1^b^	1 ± 0^b^	1 ± 0^b^
Funneliformis spp.	Brown, opaque, globose, >125 μm	E	3 ± 3	2 ± 0	2 ± 1	2 ± 0

* *S. terebinthifolius significantly higher than both plant types (H. patens, p = and B. alba) and bulk soil for Glomus spp. at the 95% significance level (p = 0.00159)*.

** *S. terebinthifolius significantly higher than both plant types (H. patens, p = and B. alba) and bulk soil for Rhizophagus spp. at the 95% significance level (p = 0.00168)*.

*Means followed by a different letter are significantly different by the Tukey HSD test at the 0.05 significance level*.

**Table 4B:** **Results of two way ANOVA and Tukey HSD ***post-hoc*** statistical analysis of the effects and interactions of sample site and plant type on the prevalence of ***Glomus*** spp. AMF spores**.

	**Df**	**Sum sq**	**Mean sq**	***F*-value**	**Pr (>F)**
Site	1	1.56	1.56	0.758	0.4094
Plant type	3	165.69	55.23	26.778	0.00159
Site:PlantType	3	8.69	2.90	1.404	0.310828
Residuals	8	16.50	1.404		
**Significant plant effect**	**Adjusted** ***p*****-value**				
*Schinus*:*Bidens*	0.001229				
*Schinus*:*Hamelia*	0.0004484				
*Schinus:*Bulk soil	0.0001803				

**Table 4C:** **Results of two way ANOVA and Tukey HSD ***post-hoc*** statistical analysis of the effects and interactions of sample site and plant type on the prevalence of ***Rhizophagus*** spp. AMF spores**.

	**Df**	**Sum sq**	**Mean sq**	***F*-Value**	**Pr (>F)**
Site	1	0.56	0.562	0.310	0.59271
Plant type	3	73.69	24.562	13.552	0.00168
Site:PlantType	3	4.69	1.562	0.862	0.49909
Residuals	8	14.50	1.812		
**Significant plant effect**	**Adjusted** ***p-*****value**				
*Schinus*:*Bidens*	0.0181				
*Schinus*:*Hamelia*	0.0025				
*Schinus*:Bulk soil	0.0025				

**Figure 3:**
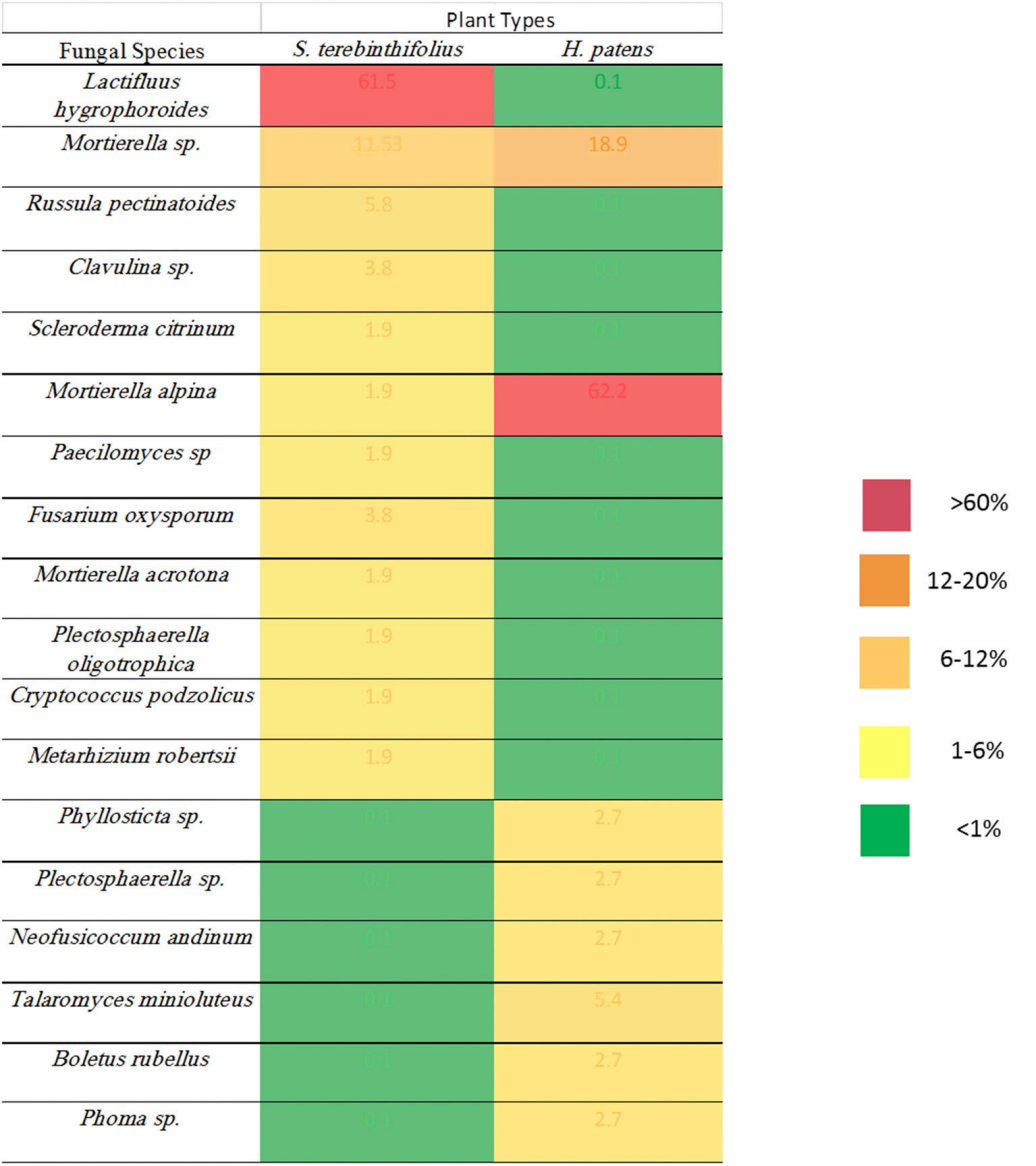
**Relative abundance of fungal species identified under the invasive ***S. terebinthifolius*** and the native ***H. patens*** rhizosphere**.

**Figure 4:**
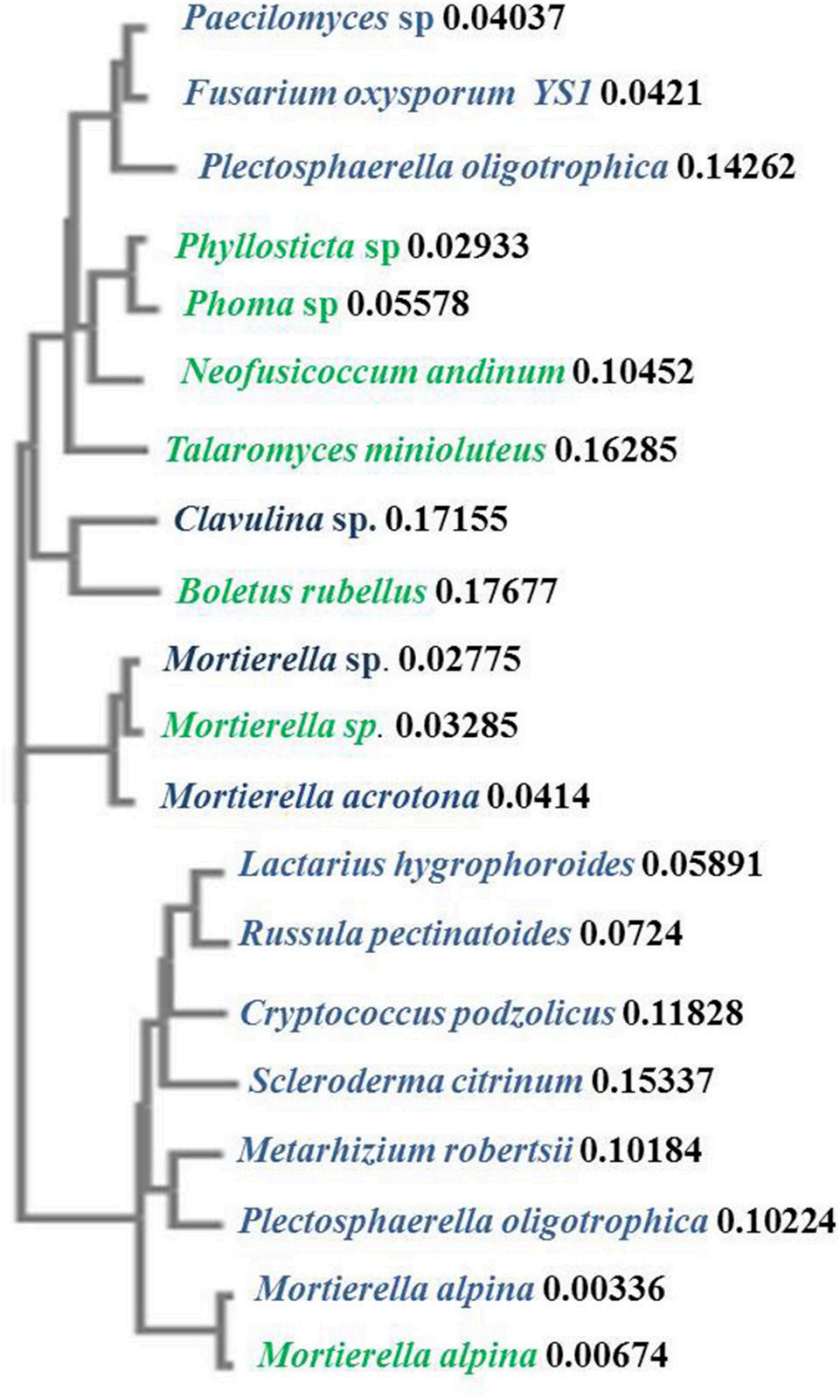
**Phylogenetic similarities between representative fungal species identified from the invasive ***S. terebinthifolius*** (blue) and the ***H. patens*** native (green) rhizosphere using the neighbor-joining clustering method**. The distance values show the number of substitutions as a proportion of the length of the alignment at the node label. The branch lengths are proportional to the amount of inferred evolutionary change.

The fungal community in the rhizosphere of a native shrub (*H. patens*) and non-native invasive shrub (*S. terebinthifolius*) growing on the same parcel of land was quite different. The ITS86F and ITS4R universal fungal primers were used to generate a ~400 bp transcript from three replicates each, of *S. terebinthifolius*, and a comparable Florida shrub—*H. patens*. Only clone-library candidates returning useful (>75% of insert and less than 10% of vector) sequences were analyzed. Three main phyla were obtained from aligned sequences, including the Basidiomycota, Ascomycota and Zygomycota fungal clades, which are the usual targets for the ITS86F and ITS4R primer set. Agaricomycetes was the most dominant class from the Basidiomycota (40.4%), while Sordariomycetes and Mucoromycotina were the most abundant from Ascomycota (25.4%) and Zygomycota (34.2%) respectively found throughout all samples. Table 5 presents the taxonomic identities, accession numbers and coefficients of similarities to closest phylogenetic relatives of fungi extracted from the test plants. Within the limits of the sample size in this study, the most prevalent (four out of six sites) in *S. terebinthifolius* rhizosphere was *Lactifluus hygrophoroides* (61.5%) (Table 5, Table S3), a member of the Basidiomycota which is a very common ectomycorrhizal fungi associated with shrubs and trees. *H. patens* soil had higher relative abundance of members of the phylum Zygomycota with saprophytic species such as *Mortierella alpina* (62.2%) from the class Mucoromycotina being the most dominant. At site 6 however both *S. terebinthifolius* and *H. patens* rhizosphere were dominated by the fungi (*Mortierella* spp.)—presumably a saprophyte. Overall, the BP rhizosphere was associated with much more ectomycorrhizae than the native shrub rhizosphere (Figures 3, 4).

**Table 5:** **Accession numbers, phylogenetic identification and functional assessment of representative fungal clones obtained from the rhizosphere of ***S. terebinthifolius*** and the Florida native shrub, ***H. patens*****.

**Plant type**	**Selected clone**	**Relative abundance (%)**	**Closest identified phylogenetic relative**	Possible^*^ function	***E*-values**	**Similarity (%)**	**Accession numbers**
***S. terebinthifolius***							
	ITS_Clone_5	61.5	*Lactifluus hygrophoroides*	Ectomycorrhizal	2E-135	93	KX816625
	ITS_Clone_136	11.53	*Mortierella* sp.	Saprotrophic	5E-172	98	KX816626
	ITS_Clone_6	5.8	*Russula pectinatoides*	Ectomycorrhizal	0	99	KX816627
	ITS_Clone_13	3.8	*Clavulina* sp.	Ectomycorrhizal	9E-145	95	KX816628
	ITS_Clone_18	1.9	*Scleroderma citrinum*	Ectomycorrhizal	9E-165	95	KX816629
	ITS_Clone_37	1.9	*Mortierella alpina*	Saprotrophic	0	99	KX816630
	ITS_Clone_38	1.9	*Paecilomyces* sp	Saprotrophic	3E-99	90	KX816631
	ITS_Clone_77	3.8	*Fusarium oxysporum* YS1	Saprotrophic/pathogenic	4E-137	97	KX816632
	ITS_Clone_107	1.9	*Mortierella acrotona*	Saprotrophic	0	97	KX816633
	ITS_Clone_117	1.9	*Plectosphaerella oligotrophica*	Unknown	4E-142	95	KX816634
	ITS_Clone_118	1.9	*Cryptococcus podzolicus*	Saprotrophic	2E-146	99	KX816635
	ITS_Clone_120	1.9	*Metarhizium robertsii*	Root endophyte	7E-155	100	KX816636
***H. patens*** **NATIVE**							
	ITS_Clone_176	62.2	*Mortierella alpina*	Saprotrophic	1E-147	100	KX816637
	ITS_Clone_189	18.9	*Mortierella* sp.	Saprotrophic	4E-113	93	KX816638
	ITS_Clone_158	2.7	*Phyllosticta* sp	Plant pathogen	3E-133	99	KX816639
	ITS_Clone_172	2.7	*Plectosphaerella* sp.	Unknown	5E-157	99	KX816640
	ITS_Clone_214	2.7	*Neofusicoccum andinum*	Plant pathogen	4E-147	100	KX816641
	ITS_Clone_231	5.4	*Talaromyces minioluteus*	Saprotrophic	9E-135	96	KX816642
	ITS_Clone_154	2.7	*Boletus rubellus*	Ectomycorrhizal	0	100	KX816643
	ITS_Clone_156	2.7	*Phoma* sp.	Plant pathogen	2E-61	81	KY228415

* *Possible function reported in literature for the fungal group (Smith and Read, 2008; Smith et al., 2011)*.

Within the limits of a small sequence size in this study, all the sites gave a high yield (80–100% sequences) of ectomycorrhizal for the BP; except sites 2 and 6 (where largely non-ectomycorrhizae fungi abound). In fact site 2 had only two sequences for BP (Table 2). On the other hand, only site 1 contained ectomycorrhizal sequences for the Florida native (12.5%). These ectomycorrhizae seem to display site specificity relative to the *H. patens* and not the BP. Whether this interesting trend will hold true in a large sample size is not clear. The proportion of ectomycorrhizal ITS sequences under BP were significantly higher than that for the native *H. patens* (*p* < 0.05) when the six sites were grouped into the three counties (Table 1).

## Discussion

Thorough understanding of the mechanisms underlying plant invasion is indispensable in the effort to coin counter-measures to a problem that is fast becoming a major global ecological challenge. One of the many ways to approach this complex phenomenon is to clearly delineate the factors and processes governing the differential susceptibilities of various natural communities to invasion by the same plant species. The invasion of BP in Florida has been much more aggressive and expansive than its spread in Texas and Hawaii (Hight et al., 2003) due in part to ecological perturbation and polyploidy (Cuda et al., 2006; Spector and Putz, 2006). However, there are scarcely any studies on the underlying microbial and molecular parts of the equation of successful *Schinus* invasion in Florida. Soil fungi are known to play an intricately important role in determining vegetation shifts and plant cover (Moora et al., 2011; Jansa et al., 2013) through intimate associations of beneficial arbuscular mycorrhizae (AMF) and or general linkage of common mycorrhizal networks (CMN). It is reasonable to hypothesize therefore that these fungi could have a pronounced impact on the biotic resistance of Florida soils. This study did not investigate the functional roles of fungi in the invasion of *Schinus*; rather it focused on answering the first and fundamental question of whether or not arbuscular mycorrhizal spores and ectomycorrhiza are consistently associated with the BP regardless of site in its non-native range. In parallel, two Florida native plants in close proximity to BP site were studied to permit a proper assessment of site effect on the results. All samples for AMF spore tests included lateral roots and rhizoplane soils.

### Arbuscular mycorrhiza associated with invasive *Schinus* and two florida native plants (*Hamelia patens* and *Bidens alba*) found on the same parcel of land

In all the six locations studied, the BP rhizosphere consistently harbored elevated levels of the AMF spores (>150 spores/g soil, Table 3A). In their study of the flooded Everglades National Park, Aziz et al. (1995) found relatively low numbers of AMF (16/g) associated with BP stands. Soil type, aerations, plant cover type and other edaphic characteristics influence the population of AMF spores (Moora et al., 2011; Jansa et al., 2013). The soils in this study, unlike the Aziz context were well aerated and could support high levels of AMF associations. Interestingly, *Schinus* rhizosphere reported nearly twice the population of AMF spores of the native plants (Figure 1, Table 3A). Bulk soil, (without plant cover), recorded the lowest average AMF spore count as expected since these micro-organisms form strong mutualistic symbiosis with plants (Jansa et al., 2013). In all cases, BP rhizosphere samples contained significantly (*p* < 0.001) higher AMF spores than the Florida natives. High AMF diversity and abundance (as reported here) is known to correlate with greater potential to protect the host plants against pathogens than a solitary AMF species can (Wehner et al., 2009).

The AMF morphotypes and their relative abundance (Figure 2, Table 4A) reveal a striking pattern where *Acaulospora* spp., the most abundant AMF in the bulk soil and the two Florida native plant samples (28–32% of total AMF) was drastically reduced to 9% under *Schinus* rhizosphere. And even though the *Glomus* spp. and *Rhizophagus* spp. were universally present, the invasive BP recorded a significantly higher density of these generalist mycorrhizal spores than other samples. The BP plants employed in this study were isolated from other plants following its invasive action. This eliminates the possibility of cross contaminations from neighboring native plants. Results reported here are in accord with previous studies (Nicolson and Schenck, 1979; Aziz et al., 1995; Moora et al., 2011) who found that *Glomus* and *Acaulospora* species were widespread in Florida soils and that *S. terebinthifolius* forms associations with the most dominant generalist AMF such as *Glomus* spp. and *Acaulospora* spp. A noteworthy data shown in Figure 2 is the apparent reduction in the relative abundance of the AMF *Acaulospora* spp (under the BP) which is widely used by Florida natives. In one Florida Everglades experiment, revegetation of land previously invaded by *Schinus* required physical removal and replacement of the top soil. Aziz et al. (1995) found that partially evacuating the top soils on which the BP thrived resulted in less mycorrhizae diversity and abundance compared to when BP soil was completely removed and replaced. *Alliara petiolata* and *Solidago canadensis* L. are two invasive plant species found to alter the arbuscular and ectomycorrhizal soil community of nearby mycorrhizal plants in their non-native range (Wolfe et al., 2008; Zhang et al., 2010). *Acaulospora* spp. were more abundant under the native plants studied here but further studies using increased sample size are warranted to identify if there exists host-specificity to those native plants and to validate AMF association with *S. terebinthifolius* and its specific roles in invasion if any. On another note, *Schinus* was shown to have one of the highest responses to AMF infection (34–62% colonization rate) compared to other pioneer tree shrubs in its native range in Brazil (Pasqualini et al., 2007). Whether the BP carried over its trait for prolific infection/association with AMF from its native range to Florida as an invasion tool was not directly examined here. However, the positive correlation between the abundance of AMF spores with sporulation rates in Bever et al. (1996), argue for the affirmative. Moora et al. (2011) also reported that most invasive plants will associate with widespread beneficial fungi present in a novel range in display of the so-called generalist invader phenomenon. Apart from the potential resource use efficiency conferred by AMF; Wehner et al. (2009) found that *Glomus intraradices* and *Glomus mosseae* have an increased tolerance for pathogenic infections and even reduced pathogen abundance on their host.

### Ectomycorrhiza and other soil fungi associated with invasive *Schinus* and two florida native plants (*Hamelia patens* and *Bidens alba*) found on the same parcel of land

In Florida, invasive BP stands are often found in mono-culture plots, having displaced weeds and other surrounding native plants. It was therefore relatively easy to collect pure and representative rhizosphere samples without contamination from other plants. Only 89 sequences out of several cloned ITS2 gene was of sufficient quality for BLAST and subsequent analysis. Notwithstanding, Figures 3, 4 and Table 5 show that the invasive *Schinus* has much higher abundance and diversity of ectomycorrhizal fungi than the Florida native shrub (*H. patens*) in 4 of 6 locations sampled. Only two sequences were obtained for BP in site 2 while 6 sequences were obtained for the native *H. patens* (Table S3). This could somewhat explain the lack of ectomycorrhiza for BP at this site.

It is known that the rhizobiome of plants is a product of plant selection via secretions and microbial response and interaction with other microbes and the plant. So, it is not unexpected that *H. patens* which is phylogenetically unrelated to the *Schinus* yielded a different group of fungi. The goal was to characterize the rhizobiome community of each plant in various sites and then test the prevalent fungal groups for site effect. And although there were no phylogenetic relatives of the *Schinus* in close proximity to the sampling sites, the experimental design was sufficient to answer the questions posed. We report here, perhaps for the first time, the abundance of ectomycorrhiza, especially members of the Basidiomycota phylum under *S. terebinthifolius* rhizosphere. *Russula* and *Lactarius*/*Lactifluus*, very common ectomycorrhizal fungi (EMF) of shrubs and trees (Gardes and Bruns, 1993; Smith et al., 2011) were most abundant (Figures 3, 4). A much larger sequence sample size would be needed to allow generalizations in terms of the precise relative abundance of the various fungal species under the BP and Firebush (*H. patens*). However, the striking differences in the proportions of ectomycorrhiza between *Schinus* and the native Florida shrub in most sites are noteworthy. Ectomycorrhiza are mostly associated with woody perennial plants (Smith and Read, 2008) similar to *Schinus*. Ectomycorrhizal fungi have been implicated in *Pinus* spp. re-establishment during invasion (Richardson et al., 1994). Ectomycorrhizal plant symbionts provide many of the same benefits as AMF such as improved water uptake and nutrient cycling (Richardson et al., 1994). The widespread presence of these common ectomycorrhizal fungi could be an indication of the prevalence of *S. terebinthifolius* in different geographical locations and perhaps sheds light on a possible mechanism for displacement of natives. This is the first study to document the association of *S. terebinthifolius* rhizosphere with EMF and underscores the need for more in-depth studies. The lack of specificity of EMF can prove advantageous to plants (Smith and Read, 2008) and possibly invasive plant species which are able to use the common mycorrhizal networks to acquire more nutrients. The formation of common mycorrhizal networks (Barto et al., 2012) could also be explored to explain the wide-reaching effects of the *S. terebinthifolius* on neighboring plants that it displaces. It is quite possible that it employs these hyphal networks for transfer allelochemicals to affect natives (Barto et al., 2011). In addition, nurse plants, which often colonize disturbed areas, could assist invasive pioneer species such as the *S. terebinthifolius* in establishing fungal associations. It must be noted however that the *Schinus* plants sampled in this study were not closely associated with other plants at the time of study. So the involvement of nurse plants in BP rhizosphere association with *Lactarius* sp. EMF, for example, remains an interesting quest.

Location 6 had the least number of invasive *S. terebinthifolius* stands (Table 1), a sandy physical soil structure and on-going chemical and physical treatment for BP control. This is the only location where ectomycorrhizal fungi were not found in the rhizosphere of the BP and where the fungal community consisted of saprophytes only. Site 6 also reported the highest average rhizosphere pH values (data not shown) compared to other sites and could indicate a possible abiotic effect as some soil fungi are not tolerant to changes in pH (Smith and Read, 2008).

A *Fusarium oxysporum* fungus from the phylum Ascomycota was also found under *S. terebinthifolius* rhizosphere (3.9%) and there are many strains which have both pathogenic and protective roles in the soil community. Other *Fusarium* spp. have been implicated as possible agents of invasion where invasive plants associate with them to exert pathogenic effects on native plants (Mangla et al., 2008; Day and Dunfield, 2016). These researchers showed that invasive plants may act as microbial pathogen reservoirs when introduced in the non-native habitat reducing the growth of native plants, shown using the invasive plant *Vincetoxicum rossicum*. The native perennial shrub, not phylogenetically related but which co-occurred with *S. terebinthifolius* had a much higher abundance of saprophytic fungi (*Mortierella alpina—*65%) than ectomycorrhiza (2.6%) with a 8.1% abundance of pathogenic fungi (*Phyllosticta* sp., and *Neofusicoccum andinum*). The surprisingly low abundance of ectomycorrhizal fungi in the rhizosphere of the native plant could be due to a lack of preference for ectomycorrhizal symbiosis by the *H. patens* native or more importantly the exact roles of these saprotrophs are unknown as these soil fungi may switch between obligate to free living fungi (Hibbert et al., 2000). Soil fungi exhibit different host shifts with newly introduced non-native plants where a pre-existing broad range fungus shifts to a more specific association leading to the host plant range expansion (Gladieux et al., 2015); a possible phenomenon for invasive plants. The saprotrophs (*Mortierella* sp. and *Mortierella alpina*) found under *S. terebinthifolius* and *H. patens* native had very similar sequences (Table 5, Figure 4) indicative of common source and perhaps similar roles under each plant species. However, two *Mortierella* sp. from both BP and the *H. patens* native did not cluster together (Figure 4) with the other *Mortierella* spp. Sequence divergence could indicate strain variations which in turn could mean different functional roles; making it difficult to assign mechanisms of action based on taxa alone. Zhang et al. (2011) found that some *Mortierella* spp. significantly improved the positive effects of AM fungi on a weed plant (*Kostelelzkya virginica*), giving these species a role beyond saprophytism. The functional roles assigned to the fungal groups in Table 5 are at best speculative (from literature). Hibbert et al. (2000) reported that some mycorrhizal or ectomycorrhizal symbionts which have diverse plant hosts have evolved repeatedly from free living saprotrophic fungi in a reversal cycle. It is therefore possible that some of the fungi designated as saprotrophs under the native plant rhizosphere may play ectomycorrhizal roles. Also, Table 5 and Figure 3 reveal an unusually low abundance of potential fungal pathogens under the Brazilian pepper tree. This observation is supported by Wiggers et al. (2005) who found abundant fungivorous mites associated with *Schinus* which function to reduce its fungal pathogen load. A similar phenomenon was shown for *Melaleuca quinquineveria* invasive plant model where pathogenic nematodes, were twice as much abundant under native species in Florida (Porazinska et al., 2007). To further validate this potential mechanism of invasion by *S. terebinthifolius*, it would be necessary to compare its rhizospheric soil fungal community in its native and non-native habitat in future studies. Such research will help evaluate the enemy release and enhanced mutualism hypotheses. To better understand the biotic resistance of Florida soils, studies of the diversity and richness of species in natural / rhizosphere soil, including the enemy release and recruitment of species (subject to edaphic conditions) should include the fungal community.

## Conclusion

This brief study, perhaps for the first time, exposes a remarkably high level and variety of AMF, ectomycorrhizal communities and very low pathogen dose in the rhizosphere of the Brazilian pepper tree regardless of experimental site. The striking disparity in the relative abundance and fungal community structure of the BP and Florida native plants opens up a previously unappreciated component of the plant invasion ecology in the State. Deciphering the roles of these fungal communities would allow a better understanding of the molecular mechanisms at play during the invasion of the Brazilian pepper tree in Florida, and could influence the direction of research for other invasive plant species.

## Author contributions

KD contributed equally to the research, drafting, and editing of the manuscript. NE is the senior author who guided the research, also contributing to research and editing of the manuscript.

## Funding

This work was supported by the Graduate Research and Inquiry Program Grant 2015, the Broward Faculty Research and Professional Support 2015 and TA support from Florida Atlantic University.

### Conflict of interest statement

The authors declare that the research was conducted in the absence of any commercial or financial relationships that could be construed as a potential conflict of interest.
