# Supplementary material for: Arbuscular and Ectomycorrhizal Fungi Associated with the Invasive Brazilian Pepper Tree (Schinus terebinthifolius) and Two Native Plants in South Florida
Source: Front Microbiol. 2017 Apr 20;8:665. doi: 10.3389/fmicb.2017.00665 (PMC5397465; doi:10.3389/fmicb.2017.00665)
Supplement: Supplementary file 1 [file Table1.DOCX]

Table S1: Arbuscular mycorrhiza spore counts per gram of rhizosphere soil in all sample replications from each plant type (*S. terebinthifolius* -ST, *H. patens -* HP, *B. alba -* BA) and bulk soil across the different sample sites (n=72)

| **Sample Sites** | **Rep 1 AMF spores/g** | **Rep 2 AMF spores/g** | **Rep 3 AMF spores/g** | **Average AMF spores/g** |
| --- | --- | --- | --- | --- |
| **ST Site 1** | 191.3 | 318.2 | 203.8 | 237.8 |
| **ST Site 2** | 284.9 | 268.2 | 205.9 | 253.0 |
| **ST Site 3** | 328.0 | 243.3 | 245.5 | 272.3 |
| **ST Site 4** | 191.3 | 164.3 | 180.9 | 178.8 |
| **ST Site 5** | 205.9 | 178.8 | 168.4 | 184.4 |
| **ST Site 6** | 176.7 | 224.6 | 208.0 | 203.1 |
|  |  |  | **Average per site** | 221.6 |
| **BA Native Site 1** | 112.3 | 156.0 | 135.2 | 134.5 |
| **BA Native Site 2** | 205.9 | 185.1 | 116.4 | 169.1 |
| **BA Native Site 3** | 199.2 | 193.4 | 187.1 | 193.2 |
| **BA Native Site 4** | 183.0 | 164.3 | 120.6 | 156.0 |
| **BA Native Site 5** | 155.6 | 147.7 | 139.3 | 147.5 |
| **BA Native Site 6** | 129.1 | 101.8 | 101.9 | 110.9 |
|  |  |  | **Average per site** | 151.9 |
| **HP Native Site 1** | 126.0 | 137.3 | 70.7 | 111.3 |
| **HP Native Site 2** | 106.0 | 95.7 | 102.2 | 101.3 |
| **HP Native Site 3** | 112.3 | 112.3 | 93.6 | 106.1 |
| **HP Native Site 4** | 118.5 | 147.7 | 185.1 | 150.4 |
| **HP Native Site 5** | 89.4 | 89.5 | 118.5 | 99.1 |
| **HP Native Site 6** | 91.6 | 76.9 | 72.7 | 80.4 |
|  |  |  | **Average per site** | 108.1 |
| **Bare Soil Site 1** | 87.3 | 52.0 | 68.6 | 69.3 |
| **Bare Soil Site 2** | 97.7 | 95.6 | 76.9 | 90.1 |
| **Bare Soil Site 3** | 72.8 | 99.8 | 101.8 | 91.5 |
| **Bare Soil Site 4** | 85.2 | 85.3 | 122.7 | 97.7 |
| **Bare Soil Site 5** | 73.0 | 60.3 | 51.0 | 61.4 |
| **Bare Soil Site 6** | 70.6 | 99.8 | 99.8 | 90.1 |
|  |  |  | **Average per site** | 83.3 |
