# Supplementary material for: Arbuscular and Ectomycorrhizal Fungi Associated with the Invasive Brazilian Pepper Tree (Schinus terebinthifolius) and Two Native Plants in South Florida
Source: Front Microbiol. 2017 Apr 20;8:665. doi: 10.3389/fmicb.2017.00665 (PMC5397465; doi:10.3389/fmicb.2017.00665)
Supplement: Supplementary file 2 [file Table2.DOCX]

Table S2: Raw data of different morphotypes of arbuscular mycorrhiza spores associated with the different plant types in the Broward county sites

|  | **ST** | | **HP** | | **BA** | | **Bulk soil** | |
| --- | --- | --- | --- | --- | --- | --- | --- | --- |
| **AMF Spore Types** | Rep 1 | Rep 2 | Rep 1 | Rep 2 | Rep 1 | Rep 2 | Rep 1 | Rep 2 |
| *Acaulospora* spp. Site 1 | 3 | 1 | 6 | 4 | 7 | 3 | 2 | 2 |
| *Acaulospora* spp. Site 2 | 6 | 4 | 8 | 5 | 8 | 4 | 3 | 4 |
| *Glomus* spp. Site 1 | 8 | 12 | 1 | 1 | 3 | 1 | 2 | 0 |
| *Glomus* spp. Site 2 | 9 | 8 | 2 | 4 | 5 | 3 | 1 | 1 |
| *Septoglomus* spp. Site 1 | 2 | 0 | 1 | 1 | 0 | 1 | 1 | 2 |
| *Septoglomus* spp. Site 2 | 4 | 2 | 0 | 3 | 1 | 2 | 1 | 0 |
| *Rhizophagus* spp. Site 1 | 7 | 6 | 3 | 1 | 2 | 2 | 2 | 0 |
| *Rhizophagus* spp. Site 2 | 4 | 8 | 0 | 0 | 4 | 2 | 1 | 1 |
| *Funneliformis* spp. Site 1 | 3 | 5 | 1 | 3 | 2 | 0 | 0 | 2 |
| *Funneliformis* spp. Site 2 | 0 | 3 | 0 | 3 | 3 | 2 | 4 | 1 |
