# Supplementary material for: Arbuscular and Ectomycorrhizal Fungi Associated with the Invasive Brazilian Pepper Tree (Schinus terebinthifolius) and Two Native Plants in South Florida
Source: Front Microbiol. 2017 Apr 20;8:665. doi: 10.3389/fmicb.2017.00665 (PMC5397465; doi:10.3389/fmicb.2017.00665)
Supplement: Supplementary file 3 [file Table3.DOCX]

Table S3: Phylogenetic identification and possible functions of fungal ITS clones obtained from the rhizosphere metagenomes of *S. terebinthifolius* and the Florida native shrub, *H. patens*. (n=89)

| Plant Type / Site | ITS Clone # | E values | Identity | Phylum | Class | Order | Genus/Species | Possible Function |
| --- | --- | --- | --- | --- | --- | --- | --- | --- |
| ST1 | 5 | 2.00E-135 | 93% | Basidiomycota | Agaricomycetes | Russulales | Lactarius hygrophoroides | ectomycorrhizal |
| ST1 | 6 | 0 | 99% | Basidiomycota | Agaricomycetes | Russulales | Russula pectinatoides | ectomycorrhizal |
| ST1 | 8 | 2.00E-130 | 93% | Basidiomycota | Agaricomycetes | Russulales | Lactarius hygrophoroides | ectomycorrhizal |
| ST1 | 9 | 1.00E-132 | 93% | Basidiomycota | Agaricomycetes | Russulales | Lactarius hygrophoroides | ectomycorrhizal |
| ST1 | 11 | 2.00E-130 | 92% | Basidiomycota | Agaricomycetes | Russulales | Lactarius hygrophoroides | ectomycorrhizal |
| ST1 | 13 | 9.00E-145 | 93% | Basidiomycota | Agaricomycetes | Cantharellales | Clavulina sp. | ectomycorrhizal |
| ST1 | 14 | 6.00E-146 | 95% | Basidiomycota | Agaricomycetes | Cantharellales | Clavulina sp. | ectomycorrhizal |
| ST1 | 18 | 9.00E-165 | 95% | Basidiomycota | Agaricomycetes | Boletales | Scleroderma citrinum | ectomycorrhizal |
| ST1 | 21 | 2.00E-130 | 90% | Basidiomycota | Agaricomycetes | Russulales | Lactarius hygrophoroides | ectomycorrhizal |
| ST1 | 22 | 3.00E-134 | 91% | Basidiomycota | Agaricomycetes | Russulales | Lactarius hygrophoroides | ectomycorrhizal |
| ST1 | 27 | 7.00E-131 | 90% | Basidiomycota | Agaricomycetes | Russulales | Lactarius hygrophoroides | ectomycorrhizal |
| ST2 | 37 | 0 | 99% | Zygomycota | Mucoromycotina | Mortierellales | Mortierella alpina | saprotrophic |
| ST2 | 38 | 3.00E-99 | 90% | Ascomycota | Sordariomycetes | Hypocreales | Paecilomyces sp | saprotrophic |
| ST3 | 51 | 5.00E-132 | 90% | Basidiomycota | Agaricomycetes | Russulales | Lactarius hygrophoroides | ectomycorrhizal |
| ST3 | 53 | 7.00E-131 | 90% | Basidiomycota | Agaricomycetes | Russulales | Lactarius hygrophoroides | ectomycorrhizal |
| ST3 | 54 | 0 | 100% | Basidiomycota | Agaricomycetes | Russulales | Russula pectinatoides | ectomycorrhizal |
| ST3 | 65 | 0 | 100% | Basidiomycota | Agaricomycetes | Russulales | Russula pectinatoides | ectomycorrhizal |
| ST4 | 66 | 2.00E-135 | 91% | Basidiomycota | Agaricomycetes | Russulales | Lactarius hygrophoroides | ectomycorrhizal |
| ST4 | 69 | 1.00E-132 | 91% | Basidiomycota | Agaricomycetes | Russulales | Lactarius hygrophoroides | ectomycorrhizal |
| ST4 | 70 | 2.00E-135 | 91% | Basidiomycota | Agaricomycetes | Russulales | Lactarius hygrophoroides | ectomycorrhizal |
| ST4 | 72 | 7.00E-131 | 90% | Basidiomycota | Agaricomycetes | Russulales | Lactarius hygrophoroides | ectomycorrhizal |
| ST4 | 73 | 4.00E-128 | 90% | Basidiomycota | Agaricomycetes | Russulales | Lactarius hygrophoroides | ectomycorrhizal |
| ST4 | 77 | 4.00E-137 | 97% | Ascomycota | Sordariomycetes | Hypocreales | Fusarium oxysporum | saprotrophic |
| ST4 | 78 | 4.00E-48 | 100% | Ascomycota | Sordariomycetes | Hypocreales | Fusarium oxysporum | saprotrophic |
| ST4 | 79 | 4.00E-92 | 92% | Basidiomycota | Agaricomycetes | Russulales | Lactarius hygrophoroides | ectomycorrhizal |
| ST4 | 82 | 1.00E-50 | 98% | Basidiomycota | Agaricomycetes | Russulales | Lactarius hygrophoroides | ectomycorrhizal |
| ST4 | 86 | 4.00E-98 | 85% | Basidiomycota | Agaricomycetes | Russulales | Lactarius hygrophoroides | ectomycorrhizal |
| ST5 | 99 | 2.00E-131 | 90% | Basidiomycota | Agaricomycetes | Russulales | Lactarius hygrophoroides | ectomycorrhizal |
| ST5 | 100 | 1.00E-133 | 91% | Basidiomycota | Agaricomycetes | Russulales | Lactarius hygrophoroides | ectomycorrhizal |
| ST5 | 101 | 9.00E-135 | 91% | Basidiomycota | Agaricomycetes | Russulales | Lactarius hygrophoroides | ectomycorrhizal |
| ST5 | 103 | 2.00E-135 | 91% | Basidiomycota | Agaricomycetes | Russulales | Lactarius hygrophoroides | ectomycorrhizal |
| ST5 | 104 | 5.00E-132 | 90% | Basidiomycota | Agaricomycetes | Russulales | Lactarius hygrophoroides | ectomycorrhizal |
| ST5 | 105 | 1.00E-132 | 91% | Basidiomycota | Agaricomycetes | Russulales | Lactarius hygrophoroides | ectomycorrhizal |
| ST5 | 106 | 1.00E-132 | 91% | Basidiomycota | Agaricomycetes | Russulales | Lactarius hygrophoroides | ectomycorrhizal |
| ST5 | 107 | 0 | 97% | Zygomycota | Mucoromycotina | Mortierellales | Mortierella acrotona | saprotrophic |
| ST5 | 111 | 9.00E-73 | 87% | Basidiomycota | Agaricomycetes | Russulales | Lactarius hygrophoroides | ectomycorrhizal |
| ST5 | 112 | 1.00E-132 | 91% | Basidiomycota | Agaricomycetes | Russulales | Lactarius hygrophoroides | ectomycorrhizal |
| ST5 | 113 | 1.00E-133 | 91% | Basidiomycota | Agaricomycetes | Russulales | Lactarius hygrophoroides | ectomycorrhizal |
| ST5 | 114 | 1.00E-132 | 91% | Basidiomycota | Agaricomycetes | Russulales | Lactarius hygrophoroides | ectomycorrhizal |
| ST5 | 115 | 4.00E-133 | 91% | Basidiomycota | Agaricomycetes | Russulales | Lactarius hygrophoroides | ectomycorrhizal |
| ST5 | 117 | 4.00E-142 | 95% | Ascomycota | Sordariomycetes | Hypocreales | Plectosphaerella oligotrophica | ectomycorrhizal |
| ST5 | 118 | 2.00E-146 | 99% | Basidiomycota | Tremellomycetes | Tremellales | Cryptococcus podzilocus | saprotrophic |
| ST5 | 120 | 7.00E-155 | 100% | Ascomycota | Sordariomycetes | Hypocreales | Metarhizum robertsii | root endophypte |
| ST5 | 126 | 4.00E-55 | 91% | Basidiomycota | Agaricomycetes | Russulales | Lactarius hygrophoroides | ectomycorrhizal |
| ST5 | 127 | 1.00E-132 | 91% | Basidiomycota | Agaricomycetes | Russulales | Lactarius hygrophoroides | ectomycorrhizal |
| ST5 | 130 | 0 | 100% | Zygomycota | Mucoromycotina | Mortierellales | Mortierella sp. | saprotrophic |
| ST5 | 132 | 1.00E-113 | 91% | Basidiomycota | Agaricomycetes | Russulales | Lactarius hygrophoroides | ectomycorrhizal |
| ST6 | 136 | 5.00E-172 | 98% | Zygomycota | Mucoromycotina | Mortierellales | Mortierella sp. | saprotrophic |
| ST6 | 137 | 3.00E-146 | 97% | Zygomycota | Mucoromycotina | Mortierellales | Mortierella sp. | saprotrophic |
| ST6 | 138 | 4.00E-173 | 98% | Zygomycota | Mucoromycotina | Mortierellales | Mortierella sp. | saprotrophic |
| ST6 | 147 | 7.00E-176 | 97% | Zygomycota | Mucoromycotina | Mortierellales | Mortierella sp. | saprotrophic |
| ST6 | 148 | 9.00E-175 | 98% | Zygomycota | Mucoromycotina | Mortierellales | Mortierella sp. | saprotrophic |
| HP1 | 154 | 0 | 100% | Basidiomycota | Agaricomycetes | Boletales | Boletus rubellus | ectomycorrhizal |
| HP1 | 156 | 3.00E-15 | 100% | Ascomycota | Dothiodeomycetes | Pleosporales | Leptosphaeria spp. | plant pathogen |
| HP1 | 158 | 3.00E-133 | 99% | Ascomycota | Dothiodeomycetes | Botryosphaeriales | Phyllosticta spp | plant pathogen |
| HP1 | 168 | 3.00E-114 | 93% | Zygomycota | Mucoromycotina | Mortierellales | Mortierella alpina | saprotrophic |
| HP1 | 169 | 0 | 100% | Zygomycota | Mucoromycotina | Mortierellales | Mortierella alpina | saprotrophic |
| HP1 | 170 | 0 | 100% | Zygomycota | Mucoromycotina | Mortierellales | Mortierella alpina | saprotrophic |
| HP1 | 172 | 5.00E-147 | 99% | Ascomycota | Sordariomycetes | Hypocreales | Plectosporium sp. | plant pathogen |
| HP1 | 176 | 1.00E-147 | 100% | Zygomycota | Mucoromycotina | Mortierellales | Mortierella alpina | saprotrophic |
| HP2 | 189 | 4.00E-113 | 92% | Zygomycota | Mucoromycotina | Mortierellales | Mortierella spp. | saprotrophic |
| HP2 | 194 | 3.00E-164 | 100% | Zygomycota | Mucoromycotina | Mortierellales | Mortierella alpina | saprotrophic |
| HP2 | 195 | 0 | 99% | Zygomycota | Mucoromycotina | Mortierellales | Mortierella alpina | saprotrophic |
| HP2 | 197 | 5.00E-162 | 100% | Zygomycota | Mucoromycotina | Mortierellales | Mortierella alpina | saprotrophic |
| HP2 | 205 | 5.00E-162 | 100% | Zygomycota | Mucoromycotina | Mortierellales | Mortierella sp. | saprotrophic |
| HP2 | 207 | 5.00E-53 | 93% | Zygomycota | Mucoromycotina | Mortierellales | Mortierella sp. | saprotrophic |
| HP3 | 214 | 4.00E-147 | 99% | Ascomycota | Dothiodeomycetes | Botryosphaeriales | Neofusicum andinum | plant pathogen |
| HP3 | 218 | 8.00E-109 | 100% | Zygomycota | Mucoromycotina | Mortierellales | Mortierella sp. | saprotrophic |
| HP3 | 219 | 3.00E-112 | 100% | Zygomycota | Mucoromycotina | Mortierellales | Mortierella sp. | saprotrophic |
| HP4 | 227 | 1.00E-121 | 100% | Zygomycota | Mucoromycotina | Mortierellales | Mortierella sp. | saprotrophic |
| HP5 | 230 | 1.00E-122 | 100% | Zygomycota | Mucoromycotina | Mortierellales | Mortierella sp. | saprotrophic |
| HP5 | 231 | 9.00E-135 | 96% | Ascomycota | Eurotiomycetes | Eurotiales | Talaromyces minioluteus | saprotrophic |
| HP5 | 232 | 9.00E-145 | 99% | Ascomycota | Eurotiomycetes | Eurotiales | Talaromyces minioluteus | saprotrophic |
| HP5 | 235 | 2.00E-160 | 100% | Zygomycota | Mucoromycotina | Mortierellales | Mortierella alpina | saprotrophic |
| HP5 | 243 | 2.00E-165 | 100% | Zygomycota | Mucoromycotina | Mortierellales | Mortierella alpina | saprotrophic |
| HP6 | 247 | 6.00E-166 | 100% | Zygomycota | Mucoromycotina | Mortierellales | Mortierella alpina | saprotrophic |
| HP6 | 248 | 1.00E-162 | 100% | Zygomycota | Mucoromycotina | Mortierellales | Mortierella alpina | saprotrophic |
| HP6 | 249 | 1.00E-163 | 100% | Zygomycota | Mucoromycotina | Mortierellales | Mortierella alpina | saprotrophic |
| HP6 | 250 | 3.00E-164 | 100% | Zygomycota | Mucoromycotina | Mortierellales | Mortierella alpina | saprotrophic |
| HP6 | 251 | 6.00E-161 | 100% | Zygomycota | Mucoromycotina | Mortierellales | Mortierella alpina | saprotrophic |
| HP6 | 252 | 8.00E-165 | 100% | Zygomycota | Mucoromycotina | Mortierellales | Mortierella alpina | saprotrophic |
| HP6 | 253 | 3.00E-164 | 100% | Zygomycota | Mucoromycotina | Mortierellales | Mortierella alpina | saprotrophic |
| HP6 | 254 | 2.00E-160 | 100% | Zygomycota | Mucoromycotina | Mortierellales | Mortierella alpina | saprotrophic |
| HP6 | 255 | 6.00E-141 | 100% | Zygomycota | Mucoromycotina | Mortierellales | Mortierella alpina | saprotrophic |
| HP6 | 256 | 1.00E-158 | 100% | Zygomycota | Mucoromycotina | Mortierellales | Mortierella alpina | saprotrophic |
| HP6 | 257 | 2.00E-175 | 100% | Zygomycota | Mucoromycotina | Mortierellales | Mortierella alpina | saprotrophic |
| HP6 | 258 | 3.00E-164 | 100% | Zygomycota | Mucoromycotina | Mortierellales | Mortierella alpina | saprotrophic |
| HP6 | 259 | 3.00E-159 | 100% | Zygomycota | Mucoromycotina | Mortierellales | Mortierella alpina | saprotrophic |
| HP6 | 263 | 8.00E-165 | 100% | Zygomycota | Mucoromycotina | Mortierellales | Mortierella alpina | saprotrophic |
